# Sensitivity to extracellular potassium underlies type-intrinsic differences in retinal ganglion cell excitability

**DOI:** 10.3389/fncel.2022.966425

**Published:** 2022-08-05

**Authors:** Andrew M. Boal, Nolan R. McGrady, Michael L. Risner, David J. Calkins

**Affiliations:** Department of Ophthalmology and Visual Sciences, Vanderbilt Eye Institute, Vanderbilt University Medical Center, Nashville, TN, United States

**Keywords:** retinal ganglion cells, excitability, potassium, physiology, action potential, glaucoma, axon initial segment

## Abstract

Neuronal type-specific physiologic heterogeneity can be driven by both extrinsic and intrinsic mechanisms. In retinal ganglion cells (RGCs), which carry visual information from the retina to central targets, evidence suggests intrinsic properties shaping action potential (AP) generation significantly impact the responses of RGCs to visual stimuli. Here, we explored how differences in intrinsic excitability further distinguish two RCG types with distinct presynaptic circuits, alpha ON-sustained (αON-S) cells and alpha OFF-sustained (αOFF-S) cells. We found that αOFF-S RGCs are more excitable to modest depolarizing currents than αON-S RGCs but excitability plateaued earlier as depolarization increased (i.e., depolarization block). In addition to differences in depolarization block sensitivity, the two cell types also produced distinct AP shapes with increasing stimulation. αOFF-S AP width and variability increased with depolarization magnitude, which correlated with the onset of depolarization block, while αON-S AP width and variability remained stable. We then tested if differences in depolarization block observed in αON-S and αOFF-S RGCs were due to sensitivity to extracellular potassium. We found αOFF-S RGCs more sensitive to increased extracellular potassium concentration, which shifted αON-S RGC excitability to that of αOFF-S cells under baseline potassium conditions. Finally, we investigated the influence of the axon initial segment (AIS) dimensions on RGC spiking. We found that the relationship between AIS length and evoked spike rate varied not only by cell type, but also by the strength of stimulation, suggesting AIS structure alone cannot fully explain the observed differences RGC excitability. Thus, sensitivity to extracellular potassium contributes to differences in intrinsic excitability, a key factor that shapes how RGCs encode visual information.

## Introduction

The central nervous system is composed of many neuronal classes and types defined by genetic, neurochemical, morphologic, and physiologic properties that determine function. This biologic complexity is apparent across neural tissues and exemplified in the retina, which is a projection of the diencephalon. Different retinal neuronal classes and types interact through synaptic and electrical signals, forming local receptive fields. Receptive field elements converge, diverge, and converge again to optimize the signal-to-noise ratio of graded potentials received by postsynaptic retinal ganglion cells (RGCs) (Cohen and Sterling, 1990; Copenhagen et al., 1990; Asari and Meister, 2012). RGCs integrate postsynaptic potentials, generate spike trains that encode sensory information, and relay action potentials with high fidelity to central targets (Uzzell and Chichilnisky, 2004; Pillow et al., 2005). The presence of a large diversity in RGC response properties underlies the ability to encode complex visual scenes (Baden et al., 2016).

Currently, RGC types are distinguished by both intrinsic and extrinsic properties, including genetic profile, transcriptomics, morphology, regular topographic spacing, and physiologic response to stimuli (Sanes and Masland, 2015; Shekhar and Sanes, 2021). Based on these variables, 20–40 different RGC types of have been identified, depending on species (Sanes and Masland, 2015; Baden et al., 2016; Bae et al., 2018; Tran et al., 2019; Goetz et al., 2022). Measuring RGC response bias to light increments and decrements has been a mainstay in RGC taxonomy, but this method tests both extrinsic influences, such as presynaptic inputs, and intrinsic mechanisms, such as threshold for action potential generation. Alternatively, evidence suggests RGC types may be physiologically defined by measuring their responses to application of hyperpolarizing or depolarizing currents (Margolis and Detwiler, 2007; Mitra and Miller, 2007b,a; Cai et al., 2011). Testing these intrinsic properties is important because inherent differences can significantly influence spiking output in RGCs, establishing different light responses in cells with similar synaptic inputs (Emanuel et al., 2017; Werginz et al., 2020; Wienbar and Schwartz, 2022).

Here, we tested physiologic differences between two RCG types with distinct synaptic inputs, alpha ON-sustained (αON-S) cells, which generate sustained firing of action potentials to light increments, and alpha OFF-sustained (αOFF-S) cells, which are inhibited by light and produce sustained firing upon light decrement. We found that these two types also demonstrate different intrinsic responses to application of depolarizing currents and correspondingly distinct AP waveforms. We also demonstrated that sensitivity to extracellular K^+^ concentrations may mechanistically underlie these differences in excitability profiles and establish thresholds for depolarization block, which is a mechanism that limits neuronal firing rate during excess depolarization due to inactivation of voltage-gated sodium channels. These results support the growing body of evidence that RGC responses are not only determined by presynaptic circuitry but also are significantly impacted by variability in intrinsic neuronal mechanisms.

## Materials and methods

### Animals

We obtained C57Bl/6J mice (6 male and 2 female, 14–17 weeks old) from Jackson Laboratories (Bar Harbor, ME, United States, RRID:IMSR_JAX:000664). Mice were housed at the Vanderbilt University Division of Animal Care and maintained on 12-h light/dark cycle. Animals were allowed water and standard rodent chow *ad libitum*. All animal experiments were approved by the Vanderbilt University Medical Center Institutional Animal Care and Use Committee.

### Electrophysiological recordings

Animals were euthanized via cervical dislocation, eyes were enucleated, and the retinas were dissected out under long-wavelength illumination (630 nm, 800 μW/cm^2^, FND/FG, Ushio, Cypress, CA, United States). Retinas were placed in carbogen-saturated Ames’ medium (US Biologic, Memphis, TN, United States) supplemented with 20 mM D-glucose and 22.6 mM NaHCO_3_ (pH 7.4, 290 Osm). Each retina was mounted flat onto a physiological chamber, inner retina facing upward, and perfused at a rate of 2 ml/min with Ames’ medium maintained at 35°C (Model TC-344C, Warner Instruments, Hamden, CT, United States).

Retinal ganglion cells were viewed under differential interference contrast (DIC) using an Andor charge-coupled device (CCD) camera attached to an Olympus BX50 upright microscope at 40×. RGCs were targeted for intracellular recording with pipettes pulled from borosilicate glass (I.D. 0.86 mm, O.D. 1.5 mm; Sutter Instruments, Novato, CA, United States) and filled with (in mM): 125 K-gluconate, 10 KCl, 10 HEPES, 10 EGTA, 4 Mg-ATP, 1 Na-GTP, and 0.1 ALEXA 555 (Invitrogen, Carlsbad, CA, United States). The intracellular solution pH was 7.35 and osmolarity was 285 Osm. Pipettes containing intracellular solution had a resistance between 4 and 8 MΩ. Whole-cell signals were amplified (Multiclamp 700B, Molecular Devices, San Jose, CA, United States) and digitized at a sampling rate of 10 kHz (Digidata 1550A, Molecular Devices, San Jose, CA, United States). Access resistance was monitored and maintained ≤30 MΩ. All recordings were performed in current-clamp whole-cell patch-clamp configurations.

During baseline recordings we measured resting membrane potential (RMP), spontaneous spiking, light-evoked spike activity (full-field 365 + 460 nm, 300 μW/cm^2^, 3-s duration, CoolLED, pE-4000, Andover, United Kingdom), and current-evoked spiking while clamping the cell at 0 pA. Current-evoked spiking was measured during stepwise application of 1 s current pulses, ranging from 0 to 300 pA in 10 pA increments, with a 2 s inter-stimulus interval. Very few αOFF-S cells exhibited sustained spiking at stimuli greater than 300 pA so we set that as the upper limit for recordings. Current was clamped at 0 pA between test pulses.

### High extracellular potassium recordings

A second batch of Ames’ medium was prepared as described above, but with an additional 5 mM of KCl (i.e., high K^+^). After baseline recordings were completed for each cell, the high K^+^ medium was washed into the recording chamber while the RGC spontaneous membrane voltage was continuously recorded. Before performing experiments, we allowed a wash on period of 5–6 min, allowing RGC membrane potential to stabilize. High K^+^ experimental recordings (RMP, spontaneous activity, light-evoked spiking, and current-evoked spiking) were then performed as described above. After high K^+^ experiments, the extracellular medium was switched back to the baseline solution, and RGC membrane voltage was continuously measured during a wash off period of 10–20 min, allowing RGCs to recover. Full recovery typically took 10–15 min, although it occasionally required up to 20 min. We limited the number of experimental protocols to reduce the time of high K^+^ exposure. Furthermore, to limit potential cumulative effects of high K^+^ wash on/off, we limited the total number of cells that were recorded from each retina to no more than four.

### Retinal ganglion cell physiology analysis

Raw electrophysiology data files were analyzed in Python 3.9 (RRID:SCR_008394) using the pyABF 2.3.5 module (Harden, 2022) and SciPy 1.7.1 modules (RRID:SCR_008058) (Virtanen et al., 2020). The SciPy.Signal “find_peaks” function was used for AP detection with specified parameters of 20 mV minimum prominence and a distance threshold of 1.5 ms between spikes. Spike rates for current-evoked spiking protocols were reported as the average rate for 2 adjacent 10 pA increments of stimulation (20 pA bins). For AP shape analysis, a cubic spline function was fit to each AP waveform to increase resolution. The AP half-width was measured as the duration of the AP, in ms, where the membrane potential was above the midway point between each AP peak and minimum after-hyperpolarization.

### Immunohistochemistry and imaging

After physiology, retinas were placed in 4% paraformaldehyde at 4°C for 24 h. After fixation, retinas were immunolabeled for choline acetyltransferase (ChAT, 1:100; Millipore, Burlington, MA, United States, Cat. #AB144P, RRID:AB_2079751) and ankyrin-G (AnkG, 1:200; NeuroMab N106/36; Antibodies, Inc. Cat. # 75–146, RRID:AB_10673030). Retinas were first blocked in 5% normal donkey serum for 2 h, then incubated in primary antibodies for 3 d at 4°C, and finally incubated for 2 h at room temperature with donkey anti-goat Alexa 647 and donkey anti-mouse Alexa 488 secondary antibodies (Jackson ImmunoResearch, West Grove, PA, United States; RRID:AB_2340437, RRID:AB_2341099). We used an Olympus FV1000 inverted microscope to obtain micrographs of RGC profiles. Image analysis, including creating orthogonal projections used for visualization of dendritic stratification depth, was performed using ImageJ (NIH, Bethesda, MD, United States). Soma size was determined in ImageJ by outlining the area encompassed by the soma in the maximum Z-stack projection from the confocal image of each dye-filled cell.

### Axon initial segment measurements

Eight αON-S and six αOFF-S cells had identifiable axon initial segment (AIS) labeling. Fluorescence of AnkG was measured from the edge of the soma along the axon of each RGC in ImageJ. Background fluorescence was subtracted from AnkG intensity profiles using a rolling ball filter with a radius of 50. Smoothed AnkG profiles were generated using a Savitzky-Golay filter with a first order polynomial fit. AIS bounds were systematically defined as the extent where smoothed ankG values were greater than 50% of the difference between baseline and maximum intensity. One αON-S AIS was excluded from analysis as its length was identified as a significant outlier by Grubbs’ Test (alpha = 0.05).

### Data analysis and statistical tests

All data are reported as mean ± standard error of the mean (SEM) unless otherwise indicated. All statistical tests were performed in GraphPad Prism 9 (Graphpad Software, San Diego, CA). All data sets were checked for normality. Where appropriate, parametric statistical tests (unpaired *t*-test, paired *t*-test, 2-way ANOVA, simple linear regression) were performed. Additional statistical analysis was done performed using mixed-effects analysis where indicated. We defined statistical significance as a *p*-value of 0.05 or less. Exact *p*-values are indicated in figure legends, alongside the specific statistical test used for each analysis.

## Results

### Intrinsic cell type-specific differences in spike rate and waveform

We targeted α-type RGCs from intact retinas for whole-cell current-clamp recording and dye filling by identifying large cell bodies. Following physiology, we recovered morphology of dye-filled cells by confocal microscopy. As before (Risner et al., 2018, 2020, 2021, 2022), we separated RGC types based on soma size, dendritic stratification within the inner plexiform layer (IPL), and light-evoked responses. Here, we focused our analysis on two well-characterized αRGCs: αON-S (*n* = 11 cells from 6 mice) and αOFF-S RGCs (*n* = 6 cells from 5 mice) (Krieger et al., 2017).

**FIGURE 1:**
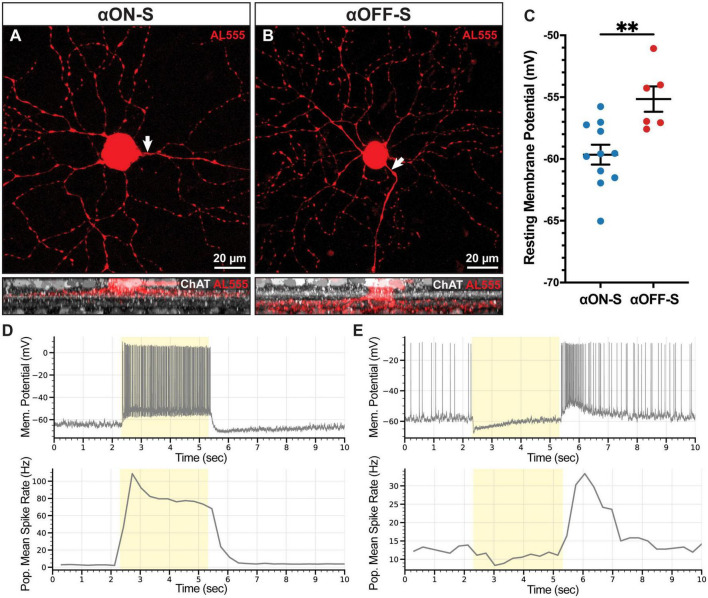
Morphologic and physiologic characterization of retinal ganglion cells (RGCs). Patched cells were filled with Alexa-fluor 555 dye (AL555, red) and morphologically reconstructed with confocal microscopy. **(A,B)** Representative maximum intensity projections of alpha ON-sustained (αON-S) and alpha OFF-sustained (αOFF-S) RGCs demonstrate characteristic soma size and dendritic branching patterns (upper). White arrows indicate the axonal projection. Orthogonal projections of representative AL555-filled cells co-labeled for choline acetyltransferase (ChAT, white) demonstrate the branching of αON-S and αOFF-S dendrites in the ON- and OFF-sublaminas of the inner plexiform layer, respectively (lower). **(C)** αOFF-S RGCs have a more depolarized resting membrane potential (mean = –55.2 mV, *n* = 6) than αON-S (mean = –59.7 mV, *n* = 11) (*p* = 0.004, unpaired *t*-test). Error bars ± standard error of the mean. **(D,E)** Representative current-clamp traces of the responses of αON-S **(D, upper)** and αOFF-S **(E, upper)** RGCs to full-field light stimulation (yellow span) show the characteristic light responses of the two cell types. Mean firing rates of all αON-S **(D, lower)** (*n* = 11) and αOFF-S **(E, lower)** (*n* = 6) binned into 200 ms intervals during light stimulation (yellow). ^**^p < 0.005.

αON-S RGCs possessed large cell bodies (307.7 ± 22.9 μm^2^) with dendritic arbors projecting within the proximal plexus formed by starburst amacrine cell dendrites, which we identified by ChAT labeling (Famiglietti and Kolb, 1976; Galli-Resta et al., 2000; Figure 1A). αOFF-S RGCs also possessed large somas (209.2 ± 16.2 μm^2^) although smaller than αON-S RGC bodies (*p* = 0.0089). αOFF-S RGC dendritic arbors extended just beyond the distal ChAT band formed by starburst amacrine cell dendrites (Figure 1B). Similar to previous reports (Risner et al., 2020), we found αOFF-S RGC RMP significantly more depolarized vs. αON-S RGCs (−55.2 ± 1.0 mV vs. −59.7 ± 0.8 mV, *p* = 0.004, Figure 1C). RMP variability was consistent with previously published results (Pang et al., 2003; Risner et al., 2018). In addition to RMP, αON- and αOFF-S RGCs produced distinct voltage-gated responses to light stimulation. In the absence of light, αON-S RGC produced few action potentials (2.56 ± 0.69 Hz), but these cells generated robust and sustained spiking during light onset (Figure 1D). αOFF-S RGCs spontaneously fired action potentials in the dark (12.61 ± 3.85 Hz), and light stimulation hyperpolarized membrane potential, reducing spiking. At light offset, αOFF-S RGCs membrane potential increased, and these cells produced a brisk sustained volley of spikes, returning to baseline firing after about 3 s (Figure 1E).

**FIGURE 2:**
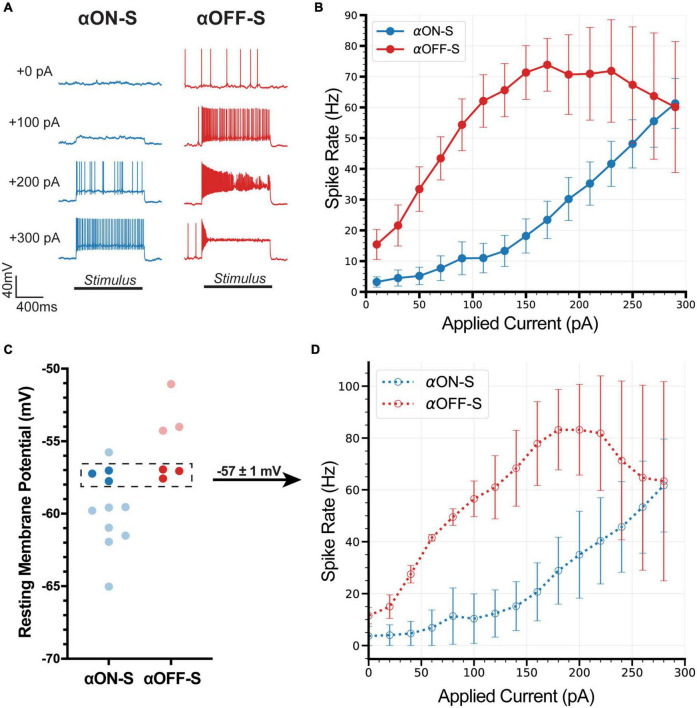
Current-evoked spike dynamics demonstrate cell type-intrinsic differences. The voltage responses of retinal ganglion cells (RGCs) were recorded during 1 s pulses of depolarizing current (0–300 pA, 2 s inter-stimulus interval). **(A)** Representative current-clamp responses of alpha ON-sustained (αON-S) (left) and alpha OFF-sustained (αOFF-S) (right) cells to 0, 100, 200, and 300 pA pulses. As magnitude of test current increases αOFF-S spike trains become more irregular and repetitive spiking is reduced. **(B)** αON-S and αOFF-S cells respond differently to the same magnitude of stimulation. αOFF-S cells fire more rapidly but ultimately plateau and decrease in rate. A two-way ANOVA analyzing the effects of cell type and depolarizing current on spike rate demonstrates a significant interaction between cell type and current (*p* < 0.0001). **(C)** To control for the influence of resting membrane potential (RMP) on current-evoked spiking we selected a subset of cells from both αON-S (*n* = 3) and αOFF-S (*n* = 3) cells with overlapping RMPs (–57 ± 1 mV) for analysis. **(D)** This subset of αON-S and αOFF-S cells still respond differently to the same magnitude of stimulation, closely resembling the pattern of the full sample **(B)**. Two-way ANOVAs indicate that there is no significant difference between the current-spiking relationship for the subset vs. full sample for either αON-S (*p* = 0.9317) or αOFF-S (*p* = 0.9618). Error bars: ±standard error of the mean.

Similar to their light responses, RGCs also produce distinct responses to hyperpolarizing current injections (Mitra and Miller, 2007a; Margolis et al., 2010; Sladek and Nawy, 2020). Here, we investigated distinguishing properties of αON- and αOFF-S RGCs in response to depolarizing currents (Twyford et al., 2014; Kameneva et al., 2016). We performed current-clamp (0 pA) recordings and measured responses of αRGCs to 1 s pulses of depolarizing current, ranging from 0 to 300 pA. We noticed αON-S RGCs typically required strong depolarizing currents to induce robust spiking (Figure 2A, left). To the counter, αOFF-S RGCs often produced a sustained train of action potentials in response to relatively small depolarizing current injections, but larger depolarizing currents often reduced spiking (Figure 2A, right). In addition to stimulus strength increasing variability in spike rate, we noted strong depolarizing currents increased variability in spike shape (Figure 2A, right). We quantified the relationship between test current and spike rate of αON- and αOFF-S RGCs. We found a significant interaction between cell type and test current (*p* < 0.0001), indicative of cell type-intrinsic differences in excitability (Figure 2B). To control for the potential influence of differing RMPs on these results (see Figure 1C), we selected a subset of cells from each type with similar RMPs (−57 ± 1 mV, Figure 2C) for comparison. We found that the current-spiking relationships of this subset closely resembled the pattern of the full sample of cells (Figure 2D), and we did not detect a significant difference in current-evoked spike rates for the subset vs. full sample for either αON-S (*p* = 0.9317) or αOFF-S RGCs (*p* = 0.9618).

**FIGURE 3:**
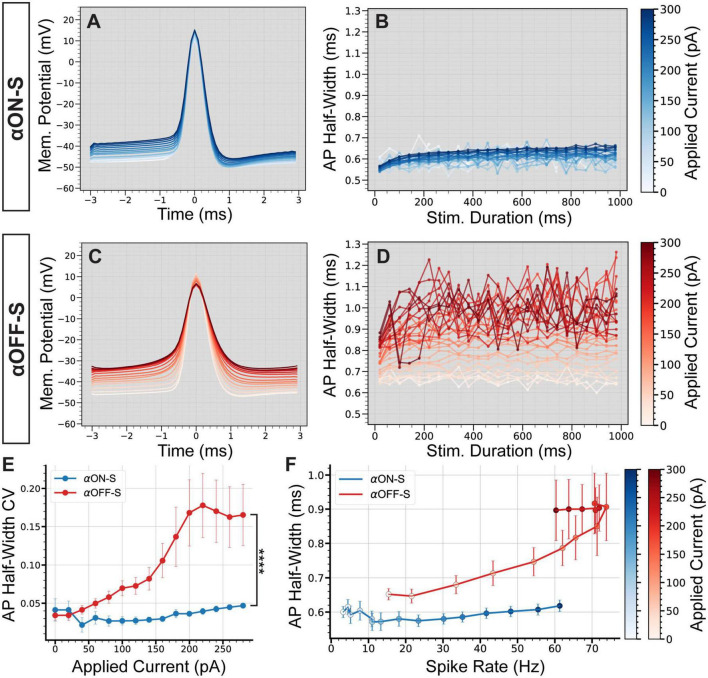
Cell type-specific differences in firing dynamics are reflected in the action potential shape. **(A)** Mean action potential (AP) shapes for alpha ON-sustained (αON-S) cells at each current step. **(B)** Mean AP half-width vs. stimulus duration for each current level of αON-S cells. **(C)** Mean AP shapes for alpha OFF-sustained (αOFF-S) cells at each current step. **(D)** Mean AP half-width vs. stimulus duration for each current level of αOFF-S cells. **(E)** AP half-width coefficient of variation (CV) vs. applied current for αON-S and αOFF-S cells. As depolarization increases, the variability of AP width increases significantly more for αOFF-S cells than αON-S (Mixed effects analysis interaction between current and cell type, *p* < 0.0001). **(F)** The mean spike rate at each current injection vs. the AP half-widths. αOFF-S retinal ganglion cells (RGCs) demonstrate significant rate-dependent spike widening (simple linear regression, non-zero slope *p* < 0.0001) while αON-S RGCs do not (*p* = 0.0668). Error bars = mean ± standard error of the mean. ****p < 0.0001.

As mentioned above, we noticed relatively small test currents appeared to impact αOFF-S RGC AP shape more than αON-S RGC APs. We determined if depolarizing currents distinctly affect αON-S vs αOFF-S RGCs by averaging AP waveforms at each test current. αON-S RGC AP waveforms remained relatively similar across test currents (Figure 3A). During the duration of larger test currents, αON-S RGC AP half-width modestly increased, though remained consistent (Figure 3B). The AP width of αOFF-S RGCs, however, grew larger with increasing depolarization (Figure 3C). Interestingly, at larger currents, αOFF-S AP half-width variability appeared greater compared to αON-S RGCs (Figure 3D). To quantify this variability, we determined the coefficient of variation (CV) of AP half-widths for each stimulus. We found as depolarization increased, the variability of αOFF-S RGC AP half-width significantly increased compared to αON-S RGCs (*p* < 0.0001, Figure 3E).

Others have found spike rate influences AP shape (de Polavieja et al., 2005). Because we found current-evoked spike rate is dependent on cell type (Figure 2), we measured the influence of spike rate on AP half-width for both αON- and αOFF-S RGCs to determine if differences in AP width could be explained solely by firing rate (Figure 3F). We found αOFF-S RGC AP half-widths significantly increased as spike rate increased (*p* < 0.0001). To the counter, αON-S RGC AP half-widths remained relatively stable as spike rate increased (*p* = 0.0668). These data suggest αON- and αOFF-S RGCs can be distinguished, in regard to AP width, by their dependence on spike rate.

### Sensitivity to K^+^ determines retinal ganglion cell type-specific differences in spike rate and waveform

**FIGURE 4:**
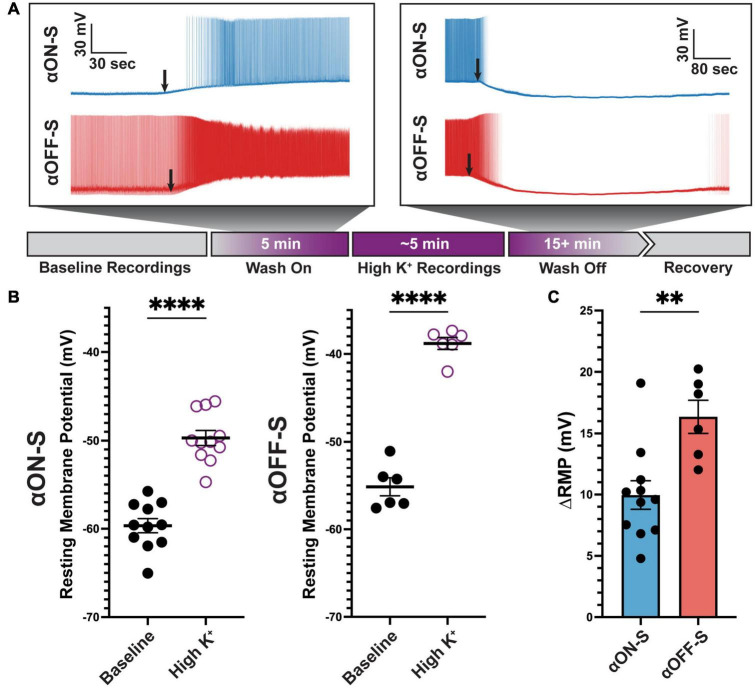
Alpha OFF-sustained (αOFF-S) cells are more depolarized by elevated extracellular potassium. **(A)** Schematic timeline of experiment, and representative spontaneous voltage responses of alpha ON-sustained (αON-S) and αOFF-S cells during high K^+^ medium (additional 5 mM KCl) wash on and off. Black arrows indicate onset of significant depolarization (left) or repolarization (right). **(B)** Resting membrane potentials (RMP) measured for each cell before and after addition of 5 mM KCl. High K^+^ significantly depolarized both αON-S and αOFF-S cells (paired *t*-tests, *p* < 0.0001 for both). **(C)** Change in RMP (ΔRMP) after high K^+^ addition. ΔRMP was significantly greater for αOFF-S cells (unpaired *t* test, *p* = 0.0038). Error bars: ± standard error of the mean. ^****^p < 0.0001, ^**^p < 0.005.

Rate-dependent spike widening is largely mediated by K^+^ currents during action potential repolarization (Ma and Koester, 1996). Based on this premise, we determined if modulating the K^+^ concentration gradient could explain cell type-specific differences in RMP, spike rate, and spike width. We employed a within-subjects approach where recordings were performed before and after bath application of extracellular medium containing additional KCl (extra 5 mM, high K^+^, Figure 4A). The additional 5 mM K^+^ in this experimental approach brought the total extracellular K^+^ concentration to 8 mM, making this treatment a physiologic depolarizing stimulus (Heinemann and Lux, 1977). High K^+^ significantly depolarized RMP of both cell types (*p* < 0.0001 for both, Figure 4B). Notably, RMP of αON-S and αOFF-S cells did not appear equally affected by high K^+^ treatment. We analyzed this apparent difference by computing the difference in baseline and high K^+^ RMP (ΔRMP). We found ΔRMP of αOFF-S cells significantly greater than αON-S RGCs (*p* = 0.0038, Figure 4C).

**FIGURE 5:**
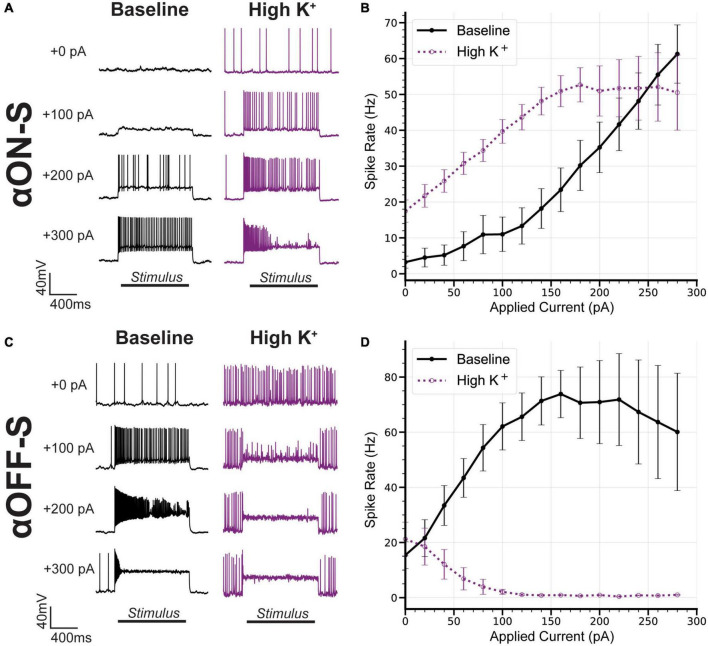
Alpha OFF-sustained (αOFF-S) firing dynamics are more sensitive to elevated extracellular potassium. **(A)** Representative current-clamp responses of alpha ON-sustained (αON-S) cells to 0, 100, 200, and 300 pA pulses, before and after washing on high K^+^. **(B)** The current-spiking relationship for αON-S before and after high K^+^. High extracellular K^+^ significantly alters the current-spiking relationship (two-way ANOVA, potassium-stimulation interaction effect *p* = 0.0397). Elevated potassium increases the firing rate of αON-S retinal ganglion cells (RGCs) at most current steps but induces a plateau and decrease in rate with variably widened and failed action potentials with stronger depolarizations. **(C)** Representative current-clamp responses of αOFF-S cells to 0, 100, 200, and 300 pA pulses, before and after washing on high K^+^. **(D)** The current-spiking relationship for αOFF-S before and after high K^+^. High extracellular K^+^ significantly alters the current-spiking relationship (two-way ANOVA, Potassium effect *p* = 0.0005). In the presence of elevated K^+^, the rates of αOFF-S cells are significantly decreased and quickly drop to zero with increased stimulation, with many failed action potentials. Error bars: ± standard error of the mean.

We next sought to determine how differences in sensitivity to high K^+^ on RMP may influence current-evoked spiking of αRGC types. We measured spiking using the same depolarizing current stimulation protocol described earlier (Figure 2). High K^+^ treatment not only increased spontaneous spiking of αON-S RGCs, but also increased spiking at smaller test currents and appeared to cause depolarization block at test currents where it previously did not occur (Figure 5A). Overall, we found high K^+^ significantly altered the test current-spike rate relationship of αON-S RGCs (*p* = 0.0397, Figure 5B). High K^+^ treatment also dramatically altered αOFF-S cell current-induced spiking. Increasing test current strength in αOFF-S cells caused depolarization block, reducing spike rate and increasing the number of failed APs (Figure 5C). Overall, we found high K^+^ significantly changed the test current-spike rate relationship of αOFF-S RGCs (*p* = 0.0005, Figure 5D).

**FIGURE 6:**
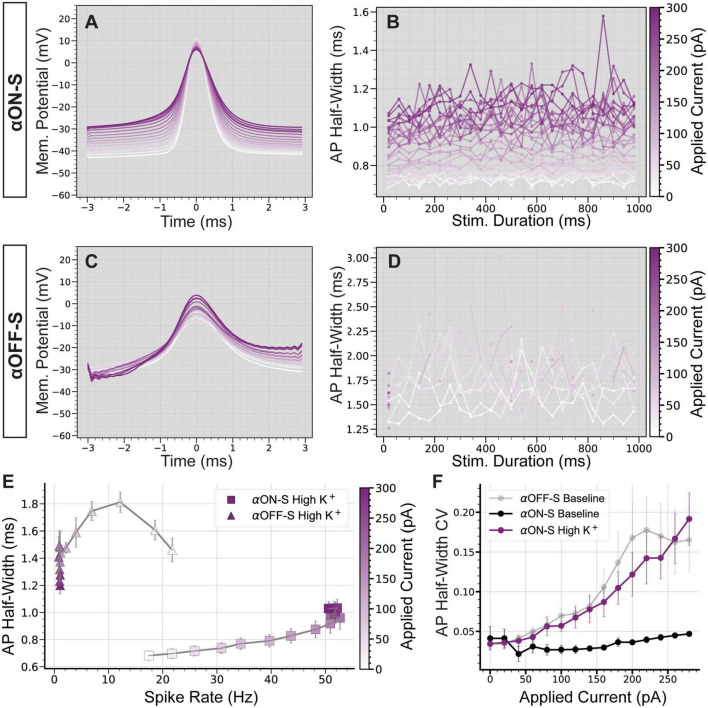
Elevated extracellular potassium significantly alters action potential shape. **(A)** Mean action potential (AP) shapes for alpha ON-Sustained (αON-S) cells in high K^+^ conditions at each current step. **(B)** Mean AP half-width vs. stimulus duration for each current level of high K^+^ αON-S cells. **(C)** Mean AP shapes for high K^+^ alpha OFF-sustained (αOFF-S) cells at each current step. **(D)** Mean AP half-width vs. stimulus duration for each current level of high K^+^ αOFF-S cells. **(E)** The mean spike rate at each current injection vs. the AP half-widths for cells in high K^+^ conditions. High K^+^ αON-S retinal ganglion cells (RGCs) demonstrate significant rate-dependent spike widening (simple linear regression, non-zero slope *p* < 0.0001). **(F)** The coefficient of variation (CV) of AP half-widths vs. magnitude of current pulse for αON-S before and after elevated K^+^, and comparison to αOFF-S baseline relationship. Mixed effects analysis of potassium and current on half-width for αON-S cells shows a significant interaction between current and potassium (*p* = 0.0253). Mixed effects analysis of half-width CVs for αON-S + high K^+^ and αOFF-S at baseline demonstrates no significant difference between the groups (*p* = 0.7190). Error bars: ± standard error of the mean.

Finally, we investigated the impact of high K^+^ on spike rate and AP shape. In addition to increasing spike rate and facilitating depolarization block (Figure 5), high K^+^ augmented AP width in αON-S cells (Figure 6A). Similar to αOFF-S RGC APs produced under baseline conditions (Figure 3D), we observed high K^+^ treatment not only increased αON-S cell AP half-width but also increased variability, especially at larger test currents (Figure 6B). High K^+^ also increased αOFF-S cell AP width (Figure 6C) and considerably decreased αOFF-S spiking, essentially eliminating sustained spiking at stronger depolarizations (Figure 6D). Interestingly, under high K^+^ conditions αON-S cells demonstrated a significantly linear correlation between spike rate and AP width (Figure 6E)—similar to αOFF-S cells under baseline conditions (Figure 3F). Furthermore, the relationship between current and AP half-width CV of αOFF-S cells under baseline conditions and αON-S cells treated with high K^+^ was not statistically different (*p* = 0.719, Figure 6F).

**FIGURE 7:**
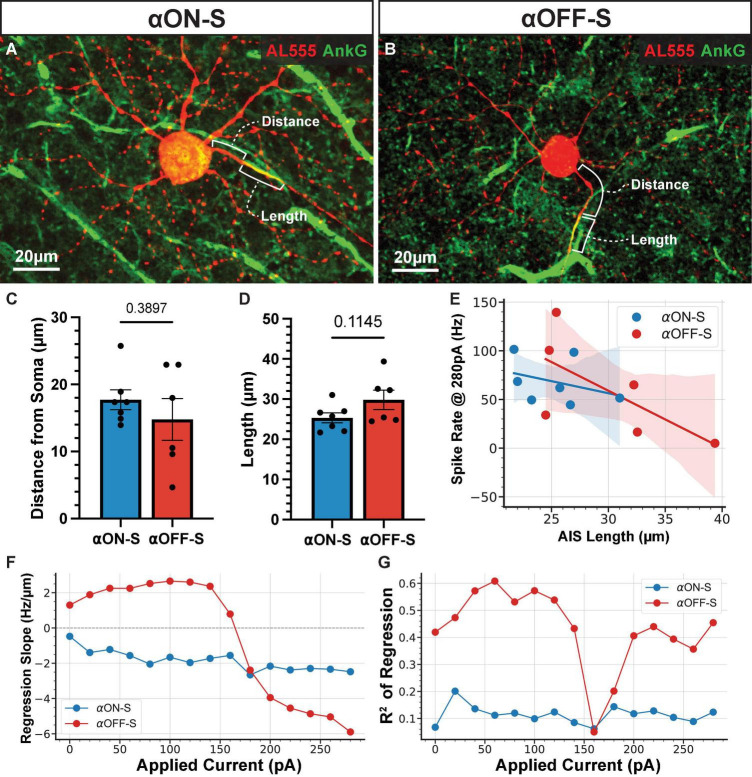
The relationship between axon initial segment (AIS) length and evoked spike rate varies by cell type and strength of stimulation. **(A,B)** Representative images of Alexa 555 (AL555, red) dye-filled alpha ON-Sustained (αON-S) **(A)** and alpha OFF-Sustained (αOFF-S) **(B)** retinal ganglion cells (RGCs) labeled for the AIS protein ankyrin-G (AnkG, green). Annotations demonstrate the dimensions of AIS distance from soma and length which are quantified below. **(C,D)** AIS distance from soma **(C)** and length **(D)** do not differ significantly between the two RGC types (*p* = 0.38397, unpaired *t*-test; *p* = 0.1145, unpaired *t*-test). Error bars: ± standard error of the mean. **(E)** Example scatter plot and linear regression best fit lines of the relationship between AIS length and current-evoked spiking rate, for 280 pA current injection. Shaded regions are 95% confidence intervals for best fit lines. **(F)** The slope of the regression best fit lines at each test current for αON-S and αOFF-S RGCs. **(G)** *R*^2^ values of the linear fits at each test current for αON-S and αOFF-S RGCs.

Evidence suggest intrinsic excitability of RGCs is in part due to AIS morphology (Werginz et al., 2020; Wienbar and Schwartz, 2022). In particular, the scaling of the AIS in α-sustained cells is important for fine tuning spiking thresholds and varies systematically by retinal topography (Raghuram et al., 2019). To evaluate the potential influence of AIS scaling on our results we labeled recorded cells for AnkG, a scaffolding protein that defines the AIS (Figures 7A,B; *n* = 7 αON-S, *n* = 6 αOFF-S). We measured AIS distance from soma and length based on AnkG immunofluorescence. We did not detect a significant difference between AIS distance from the soma (Figure 7C, *p* = 0.3897) or AIS length (Figure 7D, *p* = 0.1145) between αON- and αOFF-S RGCs. Because the length of the AIS is linked to threshold for AP generation (Jamann et al., 2021) as well as a higher threshold for depolarization block (Werginz et al., 2020; Wienbar and Schwartz, 2022) we investigated the relationship between AIS length and current-evoked spiking in our cells. We used linear regression to determine the slope of this relationship at each test current (Figure 7E). At smaller test currents (0–140 pA) αOFF-S cells had a positive correlation between AIS length and spike rate, whereas there was a moderately negative correlation for αON-S cells (Figure 7F). However, at larger test currents (180–300 pA) the relationship for αOFF-S cells flipped, with cells with longer AISs exhibiting lower spike rates. *R*^2^ values for regressions (Figure 7G) were moderately high (0.35–0.61) for αOFF-S except at test currents correlating with the onset of depolarization block (160–180 pA, *R*^2^ = 0.05–0.20), while they were fairly low at all currents for αON-S cells (0.06–0.20).

## Discussion

### Alpha ON-sustained and alpha OFF-sustained retinal ganglion cells exhibit distinct excitability profiles and spike waveforms

Our findings indicate αON-S and αOFF-S RGCs maintain distinct RMPs and voltage-gated responses to depolarizing currents, in addition to differences in light-evoked activity (Figures 1, 2). We found αOFF-S RGCs produced more robust responses to small depolarizing currents compared to αON-S RGCs (Figure 2). This difference is driven by more than simply the higher spontaneous activity of αOFF-S in these recording conditions since the slope of the increase in rate is greater for αOFF-S than αON-S cells (Figure 2B). In response to larger current injections, αOFF-S RGCs often produced few full spikes, followed by aborted action potentials, and sustained membrane potential, indicating depolarization block. To the counter, αON-S RGC responses remained robust to large current injections (Figure 2). These differences persisted even when controlling for RMP (Figures 2C,D) lending support to RGC-intrinsic mechanisms driving differences in spike rate and depolarization block. While our interpretations are limited by our sample size, these findings confirm and extend the evidence that ON and OFF RGC responses are differentially dependent on stimulus amplitude (Twyford et al., 2014; Kameneva et al., 2016).

In addition to spike rate, we found αON-S and αOFF-S RGC AP width to be differentially dependent on stimulus strength. αOFF-S RGC AP half-width dramatically increased as depolarizing current increased, whereas αON-S RGC AP half-width modestly increased in response to increasing depolarizing currents (Figures 3A–D). This AP widening phenotype closely resembles previously reported results in RGCs (Goethals et al., 2021). Stronger depolarizing currents also increased AP half-width variability in αOFF-S RGCs while αON-S RGC half-width variability remained constant across test currents (Figure 3E). αOFF-S RGC AP width variability increased prior to the onset of depolarization block (60–160 pA, Figure 2B), suggesting for an interaction between mechanisms generating depolarization block and maintaining AP half-width. We tested this notion by comparing AP half-width to spike rate for each test current (Figure 3F). We found αOFF-S RGC AP half-width significantly correlated with spike rate, but the same did not hold true for αON-S RGCs. Our findings suggest that mechanisms generating depolarization block and regulating AP half-width are related, representing a fundamental physiologic difference between αON-S and αOFF-S RGCs.

An overall limitation to this study is measurements of current-evoked responses under physiologic conditions may not completely isolate cell-intrinsic activity. Indeed, current injected at the soma will spread to both axonal and dendritic compartments. Depolarizing dendritic membranes will not only produce postsynaptic plasticity that influences voltage-gated activity, but also may induce presynaptic plasticity that can impact voltage-gated activity of the postsynaptic cell (Markram et al., 1997; Feldman, 2012). Notwithstanding, other methods for measuring intrinsic responses such as genetic models, culture systems, or pharmacology may also affect intrinsic responses.

### Sensitivity to extracellular K^+^ underlies differences in retinal ganglion cell spiking and action potential width

Rate-dependent spike widening is driven by K^+^ currents (Ma and Koester, 1996; Kasten et al., 2007). Moreover, computational modeling also links differences in K^+^ permeability to the distinct stimulation thresholds for AP failure and depolarization block in ON vs. OFF RGCs (Kameneva et al., 2016). Based on this premise, we sought to probe sensitivity to K^+^ gradients as a potential physiologic underpinning for the distinct excitability profiles and AP widths we observed in αON-S and αOFF-S cells.

Application of high K^+^ medium expectedly depolarized RMP of both αRGC types but had a significantly more pronounced effect on αOFF-S RGCs (Figures 4B,C). Similarly, the current-spiking relationship for both cell types was altered (Figure 5). αON-S cells maintained spiking activity, but additional depolarizing current input to αOFF-S cells overwhelmed spike generation capacity (Figure 5). These experiments support our key finding that αON-S and αOFF-S RGCs have different sensitivities to the K^+^ concentration gradient across their membranes. Intriguingly, the current-spiking relationship and AP shapes of αON-S cells under high K^+^ conditions closely resembled those of αOFF-S cells under baseline conditions (Figure 6). Under these conditions, αON-S cell AP failure increased and firing rate decreased with stronger depolarizing stimulation. Furthermore, the high K^+^-induced changes in αON-S AP shape at large depolarizations mirrored those seen in αOFF-S cells.

Evidence suggest AIS scaling mediates intrinsic excitability (Raghuram et al., 2019; Werginz et al., 2020; Wienbar and Schwartz, 2022). Thus, we investigated the scaling of the AIS as a potential contributor to differences in spike rate and depolarization block (Figure 7). For our sample, we did not detect significant differences in αON- and αOFF-S RGC AIS dimensions (Figures 7C,D). A limitation of this study is that we did not recover the topographic location of cells in our sample, which is important because AIS dimensions can vary with retinal topography (Raghuram et al., 2019), though we have previously reported our RGC samples are biased to the mid-to-peripheral retina (Risner et al., 2018) but without bias in respect to topography (Boal et al., 2020). Intriguingly, we found that the relationship between AIS length and spike rate varied not only by cell type, but also by the strength of stimulation (Figure 7F). At low currents, αOFF-S cells with longer AISs had higher evoked spike rates. This corroborates previous evidence that AIS length is a driver of AP generation threshold (Jamann et al., 2021). However, with stronger stimulation this relationship flipped. Beyond the point where depolarization block began to occur in the majority of αOFF-S RGCs, the cells with longer AISs had lower spike rates. Furthermore, AIS length accounted but for a small amount of the variance in αON-S cell spike rates (Figure 7G). These results support the notion that the contributions of the AIS to spiking output vary by cell type but also suggest that AIS structure cannot completely explain RGC excitability and threshold for depolarization block.

The ability of increased extracellular K^+^ to shift the response properties of αON-S toward those of αOFF-S suggests that sensitivity to extracellular K^+^ is an important component shaping the different responses of the two RGC types. There are numerous potential targets for identifying the mechanistic underpinning of this difference in K^+^ sensitivity, due to the diversity of K^+^ channels expressed in RGCs (Zhong et al., 2013). Channels associated with rate-dependent spike widening (as seen in Figures 3F, 6E) and modulating neuronal excitability, such as those mediating A-type voltage-gated K^+^ currents (Ma and Koester, 1996; Holmqvist et al., 2002), or the Ca^2+^-activated BK channels (Wang et al., 1998; Gu et al., 2007) may be promising for future investigation. RGC type-specific expression data (Tran et al., 2019; Goetz et al., 2022) present an opportunity to gain a detailed understanding of which channels may contribute to these physiological differences.

### Linking intrinsic neuronal properties to cell-type-specific vulnerabilities to degeneration

In addition to contributing to voltage-gated responses of αON-S and αOFF-S RGCs under physiologic conditions, the difference in K^+^ sensitivity between these two cells may have important implications in a pathological context, which is an ongoing topic of investigation in our laboratory. Extracellular K^+^ concentration is tightly regulated under physiologic conditions, rarely fluctuating more than a few millimolar and not exceeding 12 mM in local concentration (Heinemann and Lux, 1977). However, mechanisms regulating extracellular K^+^ concentration can become disrupted in glaucoma (Fischer et al., 2019a,b), a neurodegenerative disease affecting RGCs and their axonal projection to the brain. Furthermore, select K^+^ channels can contribute to RGC degeneration via interaction with apoptotic pathways (Diem et al., 2001; Koeberle et al., 2010). RGCs are a highly heterogeneous population, with different types defined by morphology, physiology, and molecular markers (Sanes and Masland, 2015; Baden et al., 2016; Bae et al., 2018; Tran et al., 2019). Importantly, certain cell types may be more vulnerable to glaucomatous degeneration than others, especially OFF RGCs, measured in terms of dendritic field size, arbor complexity, and excitability (Della Santina et al., 2013; El-Danaf and Huberman, 2015; Ou et al., 2016). Stressed RGCs exhibit enhanced excitability early in glaucoma, preceding frank degeneration (Risner et al., 2018, 2020; McGrady et al., 2020), and are metabolically restricted (Inman and Harun-Or-Rashid, 2017; Casson et al., 2021). Given type-specific differences in K^+^ sensitivity, dysfunctional K^+^ homeostasis in glaucoma could impose different degrees of stress on αON-S and αOFF-S cells, influencing changes to excitability, AP generation, and sustained firing, contributing to type-specific vulnerability to disease.

Broadly, understanding intrinsic neuronal properties could offer insight into the progression of many neurodegenerative diseases, which are heterogenous and affect a wide variety of central nervous system regions and cell types. Despite their disparate etiologies and pathophysiologic processes, many of these diseases share common hallmark features of progression. Notably, many diseases including Alzheimer’s Disease (Palop and Mucke, 2016), Parkinson’s Disease (Subramaniam et al., 2014), and Amyotrophic Lateral Sclerosis (Vucic et al., 2008) also exhibit neuronal hyperexcitability early in their progression. This pathophysiologic change stresses neurons, affecting metabolic demand and calcium load, pushing neurons beyond their threshold for cellular damage toward death. Further, cell type-specific vulnerability is not unique to glaucoma (Saxena and Caroni, 2011; Fu et al., 2018). Intrinsic morphologic, physiologic, and biochemical properties might underpin increased sensitivity to degeneration during stress (Fu et al., 2018). Understanding which intrinsic properties underly selective vulnerability in disease offers insight into early degenerative mechanisms and potential therapeutic targets.

## Data availability statement

The raw data supporting the conclusions of this article will be made available by the authors, without undue reservation.

## Ethics statement

The animal study was reviewed and approved by Vanderbilt University Medical Center Institutional Animal Care and Use Committee.

## Author contributions

AB, MR, and DC designed the research and wrote the manuscript. AB performed the research. AB and NM analyzed the data. All authors contributed to the article and approved the submitted version.
